# An IoT-Based Fog Computing Model

**DOI:** 10.3390/s19122783

**Published:** 2019-06-21

**Authors:** Kun Ma, Antoine Bagula, Clement Nyirenda, Olasupo Ajayi

**Affiliations:** ISAT Laboratory, Department of Computer Science, University of the Western Cape, Bellville 7535, South Africa; abagula@uwc.ac.za (A.B.); nyirendac@gmail.com (C.N.); 3944991@myuwc.ac.za (O.A.)

**Keywords:** edge computing, energy conservation, fog computing, fog layer, genetic algorithm, IoT, LIBP, multi-sink nodes, resource allocation, routing protocol, terminal layer

## Abstract

The internet of things (IoT) and cloud computing are two technologies which have recently changed both the academia and industry and impacted our daily lives in different ways. However, despite their impact, both technologies have their shortcomings. Though being cheap and convenient, cloud services consume a huge amount of network bandwidth. Furthermore, the physical distance between data source(s) and the data centre makes delays a frequent problem in cloud computing infrastructures. Fog computing has been proposed as a distributed service computing model that provides a solution to these limitations. It is based on a para-virtualized architecture that fully utilizes the computing functions of terminal devices and the advantages of local proximity processing. This paper proposes a multi-layer IoT-based fog computing model called IoT-FCM, which uses a genetic algorithm for resource allocation between the terminal layer and fog layer and a multi-sink version of the least interference beaconing protocol (LIBP) called least interference multi-sink protocol (LIMP) to enhance the fault-tolerance/robustness and reduce energy consumption of a terminal layer. Simulation results show that compared to the popular max–min and fog-oriented max–min, IoT-FCM performs better by reducing the distance between terminals and fog nodes by at least 38% and reducing energy consumed by an average of 150 KWh while being at par with the other algorithms in terms of delay for high number of tasks.

## 1. Introduction

With the wireless communication technology maturity and the progress made in the sensor/actuator and radio frequency identification technologies, the internet of things (IoT) has made its way into our daily lives by continuously growing in deployment and breakthroughs. Furthermore, the IoT terminal equipment also called sensor(s) have been continuously miniaturized, networked, and intelligently developed to support a wider range of applications in different fields. Sensors are used in an IoT infrastructure to collect data and upload it into a cloud computing facility, which has more sophisticated processors and sufficient memory resources. The amount of data being transmitted from terminal to the cloud has increased rapidly with recent increases in the number of terminal devices. This has led to issues of delay and congestion in cloud computing environments. Fog computing has emerged as a potential solution to these issues. By increasing the local computing and storage capabilities, the edge devices of the fog nodes can share a larger percentage of the processing load that would initially have been forwarded to the cloud. This in turn reduces network traffic, delays and eliminates the data storage and transmission bottleneck.

Stolfo [1], used the idea of “fog” to resist hacking. He proposed the term “fog computing”, which was later used by Cisco to promote products and networks development strategy. The concept of “fog computing” was first proposed by Cisco in 2014 [1], and defined as a distributed computing infrastructure for the internet of things (IoT) that extends computing power and data analytics applications to the “edge” of the network. A fog computing model is equivalent to a local cloud, where data management is done and controlled by the users themselves. Users can analyze and manage their data at any time, from anywhere and in any way. The core idea of fog computing lies in the “smart front-end” concepts which promotes the use of networked or dedicated devices to provide computing, storage, and network communication services between cloud servers and terminal devices. Thus, bringing data storage and computing much closer to acquisition terminal; reducing data transmission and storage overhead, improving application response speed and improving the utilization of network resources.

**Figure 1:**
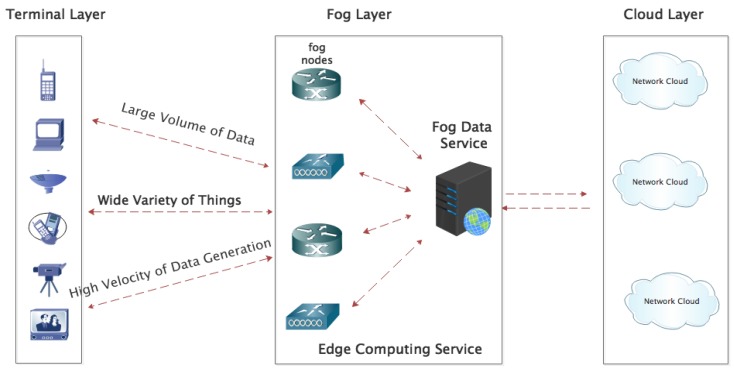
The model of the internet of things (IoT).

Fog computing can be viewed as a middle layer between cloud computing and terminal computing. It is located at the edge of the network and close to the terminal. It is often combined with cloud computing to form a common network structure model, which includes the cloud computing layer, fog computing layer and terminal access layer as shown in Figure 1. In the coverage area of the fog node, various intelligent terminals access the node and achieve interconnection and intercommunication. In  addition, the fog computing layer is able to complete the direct computing processing thereby reducing network transmission delay from sending/receiving from the remote cloud. Initially, the number of fog nodes was small and easily manageable, but their number has increased drastically in recent times. This increase in IoT terminals has brought the critical issue of energy consumption in fog nodes to the limelight. It is, therefore, an inevitably important research area for the development of IoT.

In addition, the terminal layer is a multi-hop self-organizing sensor network, composed of a large number of nodes deployed in an area and are often interconnected wirelessly. The goal is to collaboratively perceive, collect, and process information about the perceived objects in the network coverage area and send them to the observer. A large number of nodes are randomly or deterministically deployed in or near the sensor field. We assume that each node in the terminal layer has its own battery, and there are variations in power at specific time intervals due to different factors particularly the amount of activities performed by the nodes. Parameters that may affect or contribute to the degree of node overwork may include the following: traffic that can be measured in bytes or the number of child nodes that are connected to the node, ambient temperature and humidity. All of these features may result in reduced battery life. Therefore, energy consumption is always considered as the main factor in designing and measuring protocols.

With the above description, this paper proposes an IoT-based fog computing model that effectively allocates tasks to fog nodes (FN) in a manner that conserves the total energy consumed. The specific contributions of this paper are as follows:

- An IoT-based fog computing framework: this paper presents a multi-layer framework for IoT-based fog computing environments that addresses the issues related to (i) the topology of the terminal layer network and its impact on the routing of data in that layer (ii) the allocation of resources (fog nodes) in the fog layer as well as the allocation of the tasks uploaded from the terminal layer. The framework is based on a model that minimizes the overall cost (delay, distance, energy) of completing the terminal tasks using fog nodes.
- A task scheduling strategy for the fog layer: a modified genetic algorithm (GA) for the matching task (uploaded from the sink node) to the corresponding FN is proposed. The task requests and the geographical location of the fog and sink nodes are used as input for the modified GA, which outputs a binding scheme of tasks to resources (FNs). Implementation is done using CloudSim [2] and the relative efficiency of the new algorithm compared to the traditional max–min algorithm and the fog-oriented max–min algorithm [3] is revealed.
- A multi-sink model for the terminal layer: this paper proposes a novel collection tree protocol that builds upon LIBP  [4,5,6] protocol to organize the terminal layer into a multi-sink IoT network. The objective is to improve the robustness and reliability of the terminal layer network and extend the battery life of the sink nodes. Simulations using Cooja on the Contiki OS are used to demonstrate the efficiency of the multi-sink protocol compared to the mono-sink LIBP protocol.

As proposed in this paper, the IoT-based fog computing framework is aimed to complement the works done in [7,8,9,10]. Our expectation is to improve the robustness of the underlying IoT networks and safeguard these networks against nodes failures as well as extend the terminal nodes’ life span. These are achieved through the use of multi-sink deployment, while reduction in processing delays and energy consumption are achieved by incorporating a fog computing layer.

The rest of this paper is organized as follows: Section 2 introduces related works on fog computing and IoT; Section 3 describes the resource allocation model in the fog layer, while Section 4 focuses on the corresponding algorithms of fog layer model. In Section 5, the design of the terminal layer is shown, while in Section 6, experimental results and data analysis of the proposed models are discussed. Finally, conclusions and future work are presented in Section 7.

## 2. Related Work

The fog computing was first proposed by Cisco [1] in 2014. In order to solve the applicability of platform-as-a-service (PaaS), Hong et al. [11] proposed the concept of mobile fog, which realizes the connection of heterogeneous devices simplification, as well as on-demand dynamic expansion of applications, which enhances the ability to interconnect communications between heterogeneous devices and enhances the universal application of fog computing. Oueis [12] applied fog computing to the process of load balancing to improve the quality of the user’s network experience. Applications spanning cloud and fog, such as IoT applications, are still provisioned manually nowadays, but Yangui et al. [13] proposed a PaaS architecture for the automation of application provisioning in hybrid cloud/fog environment. The combination of IoT and heterogeneous devices results in utility based matching or pairing problem. This was addressed in [14] by using Irving’s matching algorithm under the node specified preferences to ensure a stable IoT node pairing. In terms of the communication distance, Intharawijitr et al. [15] defined a mathematical model of a fog network and the important related parameters to clarify the computing delay and communication delay in fog architecture. Deng [16] focused on the interplay and cooperation between the edge (fog) and the core (cloud). They developed an approximate solution to decompose the primal problem into three sub-problems to make a balance between power consumption and delay in a cloud–fog computing system. Sarkar [17] and his group conducted theoretical modelling of the fog computing architecture and analyzed the delay and energy performance of the application in the Internet of Things. They have accumulated the experience in the design and wide application of the fog computing architecture.

Due to its high degree of synthesized calculation, cloud computing cannot give full play to the resources of the edge device. Ningning et al. [18] therefore proposed a fog computing framework to turn physical nodes in different levels into virtual machine nodes. Their simulation demonstrated that a dynamic load balancing mechanism can effectively configure system resources as well as reducing the consumption of node migration brought by system changes. A lot of work in the field of task scheduling and resource management has been done [3,19,20]. In terms of the task scheduling, different computing resource has different performance and each task also has its own request, so obviously finding the best solution between task requests and computing resources is a NP hard problem. Based on this, a large number of heuristic algorithms have been proposed to find the approximate solution to the above optimal matching problem. In many application task scheduling strategies, min–min method [19] and max–min method [3] are often used as benchmark for evaluating the performance of other scheduling strategies. These two principles are similar and they are the most representative classical heuristic algorithms. For specific task scheduling problems, due to the superiority of survivability, some intelligent optimization algorithms, such as the genetic algorithm for practical scientific workflow, are also used to approximate the global optimal solution of task scheduling problem [20]. A summary of some of these related works highlighting their crux and deficiencies is shown on Table 1.

The IoT-based fog computing model (FCM) model proposed in this paper attempts to address the weaknesses of previous works.

## 3. Fog Computing Layer Design of Proposed Model

A model for the data transmission and resource allocation from underlying sensors to resources in the fog layer is proposed. The model is called an IoT-based fog computing model (IoT-FCM). As illustrated in Figure 1, it is made up of three layers, one is the terminal layer—which focuses on the IoT-driven sensors. The second one is the fog computing layer; while the last one is the cloud computing to which the fog is connected. The fog computing layer aspect of the model is described in the subsequent sub-sections, while the terminal layer of the model is discussed in Section 5. The cloud layer is outside of the scope of this paper, hence not presented.

### 3.1. The Proposed Fog Computing Model Architecture

**Figure 2:**
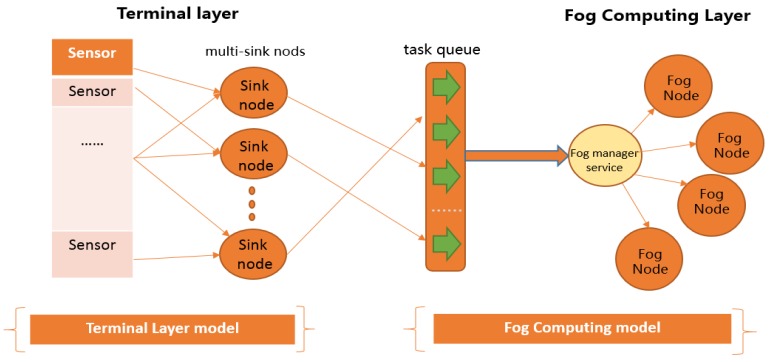
The architecture of the internet of things-based fog computing model (IoT-FCM).

Figure 2 shows the architecture of the IoT-FCM proposed in this paper. The processes performed in Figure 2 are as follows: in the first step, the application tasks queue (generated by sink-nodes) will be sent to the fog manager service which is in the fog computing layer. This service manager has information about all the fog nodes. Using the modified GA algorithm (which will be introduced in next section), the fog manager service generates the task scheduling simulation results. The FNs then executes the manager assigned tasks. Before introducing the specific process of task scheduling strategy of the modified GA for fog computing layer, some definitions and assumptions need to be introduced and this is done in Section 3.2.

### 3.2. The Proposed Optimization Framework for IoT-Based Fog Computing Environments

The fog computing layer model, which is constructed in this paper, focuses on three target parameter that decide it comprehensive performance. These parameters are: delay, energy and distance. Delay means the response time that users (sink nodes) have to wait after they submit their tasks. Energy is the total energy the target FN needs to finish its allocated tasks, while distance means the total distance of each user to their corresponding FN according to scheduling result. Suppose the fog computing system consists of the fog nodes, which can be represented as a set $FN=FN_{1},FN_{2},\ldots ,FN_{N}$, and the application tasks which are going to be scheduled can be represented as a set $T=t_{1},t_{2},\ldots t_{n}$. Delay is the main factor which can affect execution time $ExeT_{ij}$, where i = 1, …, n and j = 1, …, N. $ExeE_{ij}$ is the energy consumption of $t_{i}$ by $FN_{j}$.

The first quality factor considered is the total distance TD, which is the distance from users to their corresponding FN. This can be calculated by traversing all the tasks in set T. If ($T_{iX}$, $T_{iY}$) and ($FN_{jX}$, $FN_{jY}$), denote the coordinates of user *i* and $FN_{j}$, respectively, then TD can be determined by using

(1) $$TD=\sum_{i=1}^{n}\sqrt{{\left(T_{iX}-FN_{jX}\right)}^{2}+{\left(T_{iY}-FN_{jY}\right)}^{2}},TD<TDL,$$

where *T* is the task set, n is the number of tasks in set *T*, while connected to the *j*-th FN, and TDL is the total distance limitation.

From the fog computing characteristics, delay should be kept as low as possible. Task scheduling strategy therefore must aim at minimizing task completing time (execution time). FNs can hold more than one task at a time, the completing time is thus the execution time of such a task running on a FN whose execution time is the longest. The execution time ExeT of a task *T* by the FN can be described by using

(2) $$ExeT=\max\sum_{i=1}^{n}ExeT_{ij};j\in N,ExeT\left(T,FN\right)<DL,$$

where DL is the delay limitation, the summation of all the execution times $ExeT_{ij}$ of the various tasks $T_{i}$(i∈n) running on a FN gives the completion time of each $FN_{j}$, j∈N. The delay is obtained from the last $FN_{j}$ to finish its tasks.

Energy saving is also a very important factor that needs to be considered while building fog computing models. Therefore, fog computing system should keep the energy consumption as low as possible. In addition, the scheduling energy consumption ExeE cannot be greater than the upper limit of electricity supply. The energy consumption for executing task set *T* by set FN is given by

(3) $$ExeE=\sum_{i=1}^{n}\sum_{j=1}^{N}ExeE_{ij},ExeE\left(T,FN\right)<EL,$$

where $ExeE_{ij}$ is the energy consumed by $FN_{j}$, j∈N to execute task $T_{i}$, i∈n and ExeE is the energy consumed by all the FNs in executing their allocated tasks; EL is the energy limitation.

The three equations can be integrated into a fitness function which is defined by using

(4) $$F\left(C\right)=\left\{\begin{array}{c} \alpha *\left(1-\frac{ExeT(C)}{DL}\right)+\beta *\left(1-\frac{ExeE(C)}{EL}\right)+\gamma *\left(1-\frac{TD(C)}{TDL}\right),\\ \mathrm{if}\;\mathrm{ExeT}(C)\leq \mathrm{DL},\mathrm{ExeE}(C)\leq \mathrm{EL},\mathrm{TD}(C)>\mathrm{TDL}\\ 0,\\ \mathrm{if}\;\mathrm{ExeT}(C)>\mathrm{DL}\vee \mathrm{ExeE}(C)>\mathrm{EL}\vee \mathrm{TD}(C)>\mathrm{TDL} \end{array}\right.,$$

where C is the vector of the special individual which includes one match between tasks and fog nodes (T,FN), F(C) is the fitness function means the fitness value of the vector C, which is used for measuring the score of the individual in the population, (1 − ExeT(C)/DL) is the benefit of execution time which is considered as delay in the paper when finished the task scheduling, so the same (1 − ExeE(C)/EL) is the benefit of energy and (1 − TD(C)/TDL) is the benefit of distance. While α, β, and γ are the weight factors to adjust the importance of delay, energy consumption and distance.

## 4. The Modified Genetic Algorithm for Proposed Fog Layer Model

As mentioned earlier, task scheduling in the cloud computing environment is an NP-hard problem. It is very difficult to find the best solution when the number of participants is big. The usual way is to apply various intelligent optimization algorithms to approach its optimal solution as the satisfactory solution. A genetic algorithm is one of these algorithms to get the approximate optimal solution, and in this paper, the genetic algorithm is modified by using a single fitness function, emanating from multiple fitness functions, as well as the generation of the third child of crossover in order to determine the optimal solution of the IoT-FCM model.

The modified genetic algorithm is presented as follows:

1. Initialization of the population
  Firstly, initialization of the population and setting up of the relevant parameters, such as population size (P), probability of performing crossover (pc), probability of mutation (pm), as well as the evaluating fitness of every individual in the population are done. In genetic algorithm, the proposed multi-target parameters correspond to the multi-fitness. We use the Equation (4) as the fitness function to evaluate each vector, which in this genetic algorithm formulation is defined as a chromosome.
2. Crossover
  Following the principle of higher fitness is better, the second step involves choosing two individuals from the population as parents, upon which the crossover is executed to produce two children. In order to obtain an optimal solution, this paper adds the third child to increase the diversity of the population which is generated by accumulating the parents corresponding gene values to generate a new child. The process is showed in Algorithm 1:

**Algorithm 1:** Using two n-dimensional arrays (C1, C2) to represent two randomly selected parent chromosomes. **input**:  C1[n] = (c11, c21, …, cn1), C2[n] = (c12, c22, …, cn2) **output**:  C (Third child)[n] **1 for**
*i = 1; i ⩽ n; i++*
**do** **2** | C (Third child) [n] = (C1[n]+ C2[n])/2 **3 end**

3. Mutation
  There are many types of mutations such as Gaussian, uniform mutation and non-uniform mutation [26] and so on. In these mutations, the value of only a single gene in the chromosome is changed to improve its fitness. The effect of this on the entire chromosome is minimal especially with large population size or when the solution is close to stability [26]. We modified the mutation process, changing the single-gene mutation to multi-gene mutation. We then generate multi-mutated chromosomes to replace of the chromosomes with the lowest fitness value in the population. This reduced the impact on optimal values, while greatly expanding the search range and simultaneously reducing premature convergence in a local optimal solution. The main purpose of mutation is to generate new genes when inheriting from parents. The mutation can be defined in Equation (5) as follows:
  (5) Cm1[n]=(c1,c2,…,cn)+(x1(Δc1-c1),x2(Δc2-c2),…,xn(Δcn-cn)),
  where x1,x2,…xn∈{0,1},andΔc1,Δc2,…,Δcn are the random numbers within the limits of gene in the chromosome. Then we can generate four different children by adjusting the number of x. The first mutated child has 1/4 of its genes (x) randomly set to one, while the other genes are set to zero. The second mutated child has 1/2 its genes randomly set to one and the others set to zero. The third mutated child has 3/4 randomly set to one while the fourth has all its genes set to one.
  Since we have four mutated children, we then select four chromosomes with the smallest fitness value from the population and compare with the fitness values of our four newly generated mutated children. After the comparison, we put four chromosomes with the highest relative fitness value back into the population to get a new population. The process is showed in Algorithm 2.

**Algorithm 2:** Mutation.

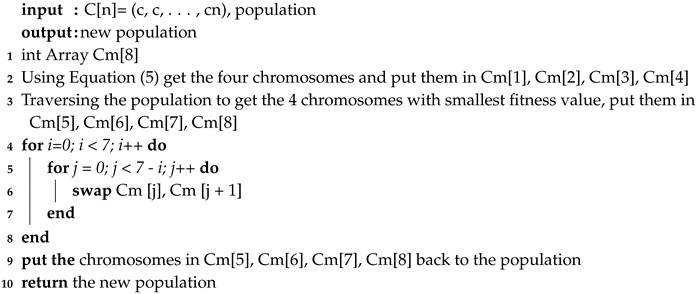

4. Merging
  In this phase, we merge the new chromosome population set generated by the crossover and mutation operations. Afterwards, the best chromosomal individuals which have the highest value of F(C) are select to be retained as population for the next generation.
5. Steps 2–4 are repeated until the simulation ends.

## 5. Terminal Layer Design of Proposed Model

**Figure 3:**
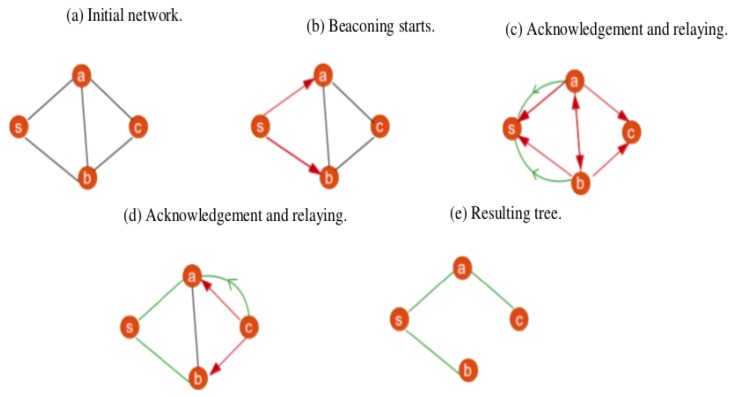
LIBP protocol description.

In this section, the second part of the IoT-FCM model which focuses on the design of the terminal layer is presented. A modification of LIBP [4,5,6] is proposed and used as a multi-sink communication protocol. As revealed by [4], LIBP is a frugal routing protocol with good performance in terms of energy consumption. It is based on a beaconing process illustrated by Figure 3 where it can be seen that i) beacon messages are used to build a spanning/collection tree rooted at the sink of the sensor network (node s) and ii) the beaconing process is implemented recursively until a spanning tree is built by broadcasting beacons for children discovery and unicasting beacons to acknowledge and select parents. The principle behind LIBP is to solve a local optimization problem by using a weight associated with a measure of interference (using number of children) in order to get a balanced tree. This is implemented by having each node below the sink node select the parent node with the fewest number of child nodes.

**Figure 4:**
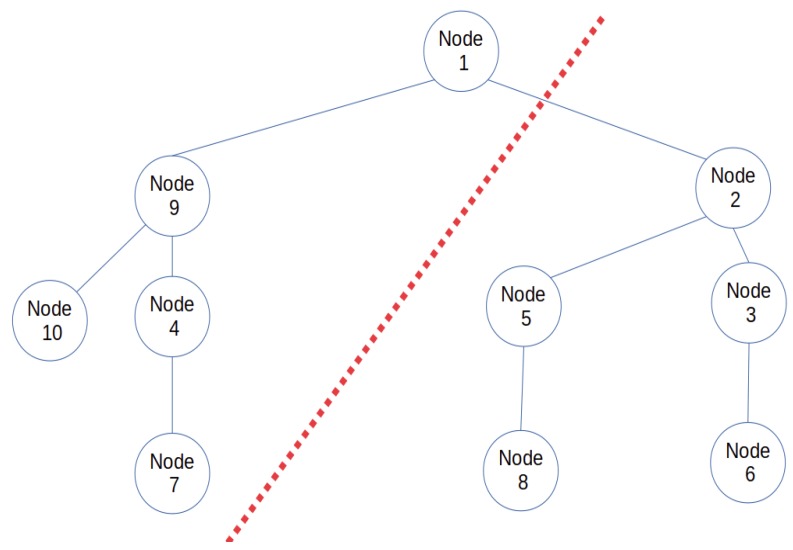
Sensor network illustration.

**Figure 5:**
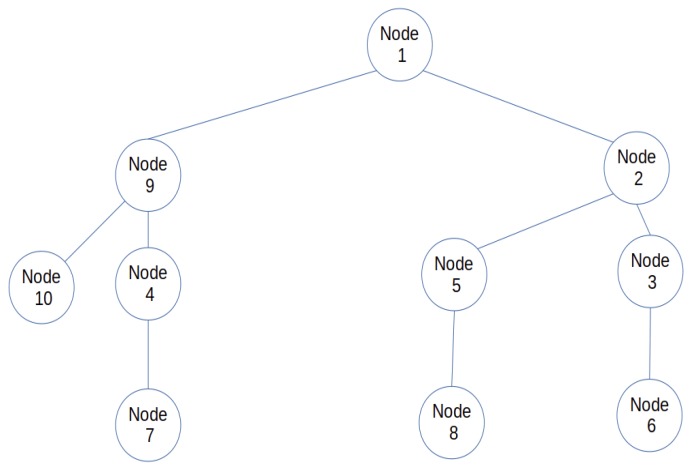
Mono sink sensor network.

**Figure 6:**
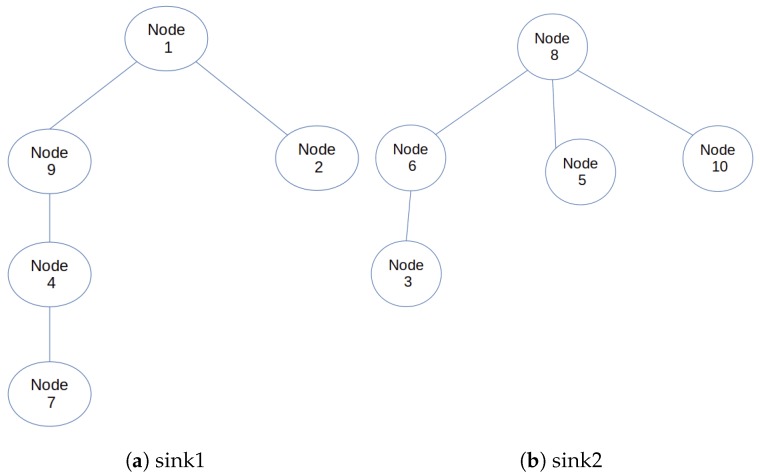
MultiSink sensor network illustration.

Though efficient, there is still room for improving the LIBP protocol, such as in areas of energy consumption and robustness of nodes when deployed in a cloud environment. Especially as nodes need a lot of energy when they communicate with nodes at greater distances. Also if the single sink node fails or is offline, the entire IoT infrastructure stops working. The multi-sink version of the LIBP proposed in this paper attempts to solve these limitations by adding multiple sink nodes to reduce the pressure on the unique sink node. The use of multiple sink nodes extends the sink nodes’ battery life and improves the robustness of the entire system. Figures 5 and 6 illustrate respectively a mono-sink sensor network and a multi-sink sensor network derived from the sensor network presented in Figure 4. These figures reveal that (i) besides the failure of the unique sink node (node 1) which can lead to the whole infrastructure stop functioning, the failure of node 2 in the mono-sink sensor network in Figure 5 will lead to partitioning the sensor network into two parts with more than half of its nodes (five nodes) failing to report data to the sink in the multi-sink configuration (ii) the multi-sink sensor networks shown in Figure 6 have a higher potential to prolong the lifetime of the sensor network, by having each non-sink node carry less children. This reduces energy consumption and limits the effect of node failure since only a maximum of 3 nodes will be cut off. For instance, in Figure 6a only three nodes will fail to report their data to node 1 if node 9 fails. Furthermore, if one of the two sink nodes (node 1 for example) fails, is attacked or is offline, the multi-sink version of the LIBP protocol, herein referred to as least interference multi-sink protocol (LIMP) will connect the orphaned nodes to the working sink node (node 8). It thus presents higher availability and robustness to failure since only part of the network may fail when a sink node is attacked or damaged and the multi-sink protocol LIMP can make the child nodes of the failed sink discover and connect to the alternative sink.

**Figure 7:**
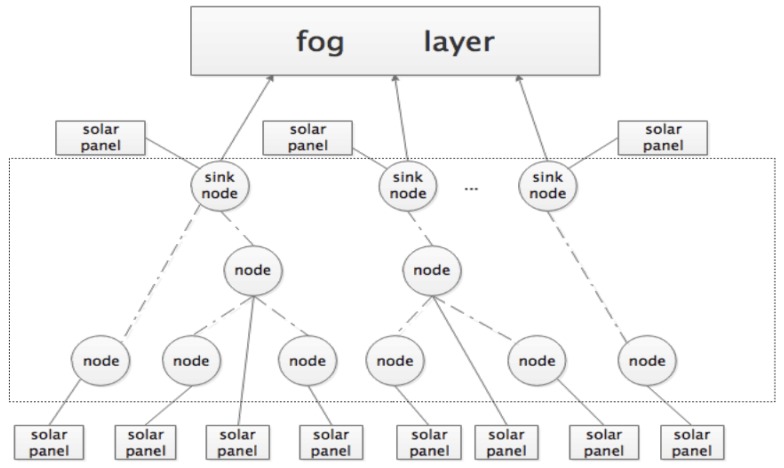
Proposed terminal layer model.

Figure 7 presents the detailed structure of the terminal layer on the left sides of Figure 2.

The model also follows several rules which are as follows:

1. There must be at least two nodes with GPRS (General Packet Radio Service). That is the terminal layer should have at least two nodes capable of transmitting IP packets to a fog node.
2. Only nodes with GPRS can become sink nodes. The sink node is selected based on whether the node has the lowest temperature and highest energy.
3. Each node should have a solar panel for energy regeneration.

## 6. Experiment and Discussion of Results

In order to evaluate the proposed IoT-FCM model, simulations of both the fog computing layer and terminal layer were done.

### 6.1. Simulation of the Fog Layer

Experimental simulations were done by extending CloudSim [2] to simulate the fog layer model proposed in this paper. CloudSim is based on the existing Java based discrete event simulation package in Grid Sim, and is developed on the existing architecture of Grid Sim. CloudSim can run on multiple platforms such as Windows and Linux. With respect to the simulation environment used in this paper, a system running Windows 7 OS with the following hardware configuration was used: CPU-Intel Pentium Dual core P6000, clocked at 1.87 GHz, 8.0 GB of memory and 1 TB of hard disk storage. In this study, eight fog nodes were used in CloudSim environment, similar to that used in [27]. Performance configuration and computing power parameters were adapted from CloudSim and are shown in Table 2.

In the simulator, the application task parameters include task ID, task length and coordinates, in which task length used millions of instruction (MI) as a unit. Task length means the number of basic instructions of task scheduling requests. For this work, task lengths were set to 1000, which was adapted from the work of [28]. We simulated the coordinates of FNs in an area, such as a city or a university, so we limited the range of FN in 0–100, and randomly generated the coordinates of 100 fog nodes, which are shown in Table 3.

Genetic algorithm operational parameters similar to those used in [27,29,30] were also used in this paper. The parameters used were as follows: a population size of 100; mutation probability of 0.01; maximum iteration number of the algorithm was set to 1000; weighting factors set as: α = β = γ = 1/3; delay limitation, energy limitation and distance were respectively set to 50, 2000 and 5000.

In this section, we show the simulation experiment results of fog computing layer in: delay (makespan), sum of distance, sum of energy consumption. Then we proved the effectiveness of our proposed GA optimized IoT-FCM model by comparing it with traditional max–min algorithm and improved fog-oriented max–min algorithm. In task scheduling problem, the traditional max–min algorithm usually select the makespan as the main parameter to achieve the relative optimal solution. The fog-oriented max–min algorithm as used in this paper considers multiple parameters (including delay, distance and energy consumption) to calculate the relative optimal solution.

#### 6.1.1. Processing Delay

**Figure 8:**
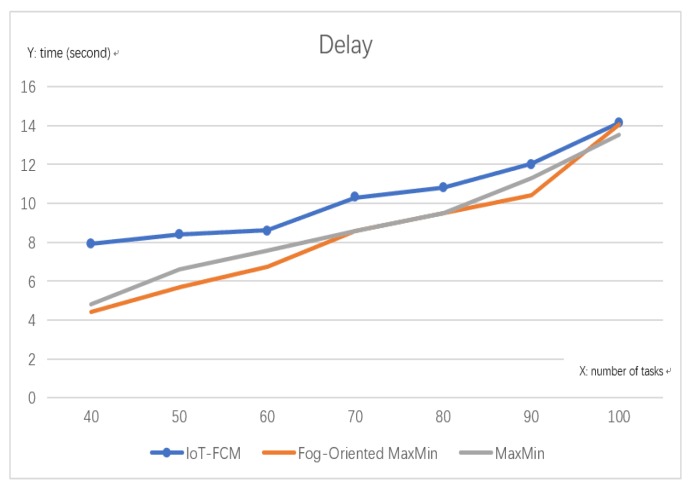
Delay.

Figure 8 shows the delay of the three different algorithms. In this paper, the delays from signal transmission time were not considered because they are too short thus negligible; rather the focus was on task execution time at the fog nodes. The proposed algorithm is aimed at minimizing the distance and energy consumption. In Figure 8, when compared to the two other algorithms (fog-oriented max–min and max–min), IoT-FCM scarified the time of delay by an average of 17.5%, since its aiming at achieving the obviously better performance in distance and energy. However, when a 100 or more tasks were submitted, it was on par with the other algorithms, as shown by the converged curves in Figure 8.

#### 6.1.2. Distance to FNs

**Figure 9:**
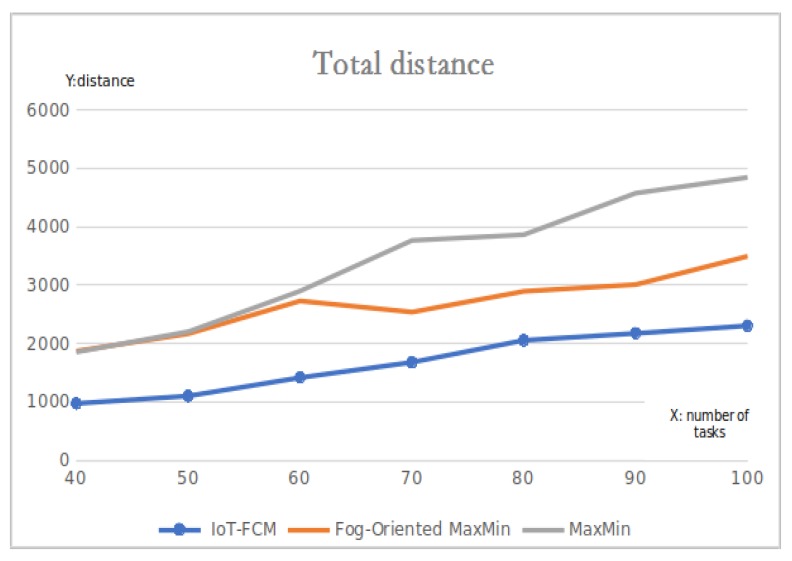
The sum of the distances from users to their corresponding fog node.

Figure 9 shows the results for the total distance form users to their corresponding FNs. One of the benefits of fog computing relative to cloud computing is that it is closer to the terminal [31]. Hence minimizing the distance is vital. From the results, comparing IoT-FCM with the two other max–min algorithms, IoT-FCM is seen to have an advantage over the others for all submitted tasks as the lower the distance between terminals and corresponding FNs, the better the algorithm. Comparing the closeness of terminals to their FNs; using IoT-FCM, terminals are about 50% closer to their FNs when 40 tasks are submitted versus the two other algorithms and 38% closer versus fog-oriented max–min and 55% closer versus max–min when 100 or more tasks are submitted. The mildness of the curve also proved the stability and predictability of the IoT-FCM model.

#### 6.1.3. Energy Consumption

**Figure 10:**
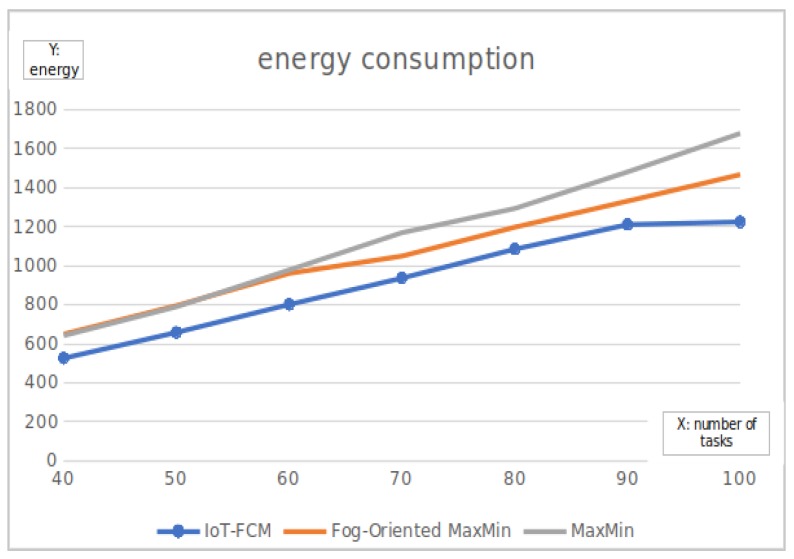
The sum of the energy consumption of all fog nodes.

Another important factor of fog computing considered in this work is the energy consumption which is shown in Figure 10. The energy consumption of all the fog nodes in the system was taken into consideration. Obtained results show that the proposed algorithm has some advantages in terms of energy consumption during the whole test. On the average, IoT-FCM conserved about 100 mAh of energy compared to fog-oriented max–min for all submitted tasks and about 200 mAh when compare to the max–min algorithm.

### 6.2. Simulating the Terminal Layer

Experiments were also carried out to simulate the terminal layer using the Cooja emulator on Contiki [32]. Cooja is a simulator/emulator embedded in Ubuntu 16.04 operating system. Using Cooja [33] we were able to implement and test the robustness and energy efficiency of the multi-sink LIBP used at the terminal layer. The experiments were run on a network with 50 nodes. When the radio chip hardware was turned on, but in passive mode (that is, not transmitting or receiving), the energy consumption was almost the same as in the receive mode. Though in passive mode, the energy consumed was not zero, because keeping the receive-machinery active and continuously sampling the medium for a potential packet transmission also consumed energy. For this experiment, the energy consumption test was done with various numbers (between 1 and 5) of sink nodes.

#### 6.2.1. Energy Consumption

From the perspective of the IoT terminal layer, conservation of the nodes’ energy consumption is the goal that the routing protocol needs to achieve. We modified the original LIBP [4], with the use of multiple Sink nodes. The simulation results have been summarized in Table 4. The table shows energy consumption levels when the modified LIBP (with multiple Sink Nodes) are used as proposed by IoT-FCM. It can be seen that the highest energy consumed by the sink node in each network are respectively 5.84%, 5.71%, 6.00%, 5.86%. These are lower than the energy consumption of 6.60% recorded when the original LIBP (with a single Sink Node) was used. Similarly, the average energy consumed when using multiple sink node in each network are respectively 4.23%, 3.92%, 4.38%, 5.07%. While the original LIBP shows that on the average, using the single sink node, energy consumption was at 6.60%. Comparing the results, the LIMP protocol used by IoT-FCM shows lower energy consumption versus the original LIBP. This in turn implies better battery life of sink nodes.

#### 6.2.2. Fault-Tolerance/Robustness

It is important to note that the energy savings and the number of sink nodes in Table 4 are not linearly related. This is because LIMP does not have to use every sink node especially if the chosen sink node is not suitable. Such is the case of the 5 sink nodes network and the three sink nodes network, where some of the sinks are orphan nodes with no children. However, though being found inadequate by the algorithm during resource allocation, the orphan nodes can be used as recovery sinks upon failure due to the multi-sink deployment. They can thus be used to improve the robustness and fault-tolerance of the terminal layer. In the simulation, we calculated the longest distance from leaf nodes to each sink nodes in different multi-sink network configurations with the goal of assessing if the distance in number of hops between two nodes can affect the delay. We also evaluated the fault-tolerance capability of the LIMP protocol by measuring the recovery time of the network upon failure of a sink node. In our case, the same sink node 1 was set offline to mimic a failure and the recovery was triggered by LIMP to migrate the orphaned nodes to alternate sink node(s). The results are shown in Table 5 reveal that multi-sink deployment does not necessarily reduces maximum distances in terms of number of hops in the terminal layer as this depends on the topology of the sensor network and the design of the LIMP protocol. However, multi-sink deployment using the LIMP protocol reduces the recovery time upon a single sink node failure.

## 7. Conclusions and Future Work

Combining the internet of things and fog computing, this paper proposed an IoT-based fog computing model and described the model in layers. The model, called IoT-FCM, is made up of two parts, in the fog computing layer part, a modified genetic algorithm was used that comprehensively considers the delay, the distance between the fog nodes and the users, as well as the energy consumption of the fog nodes to match and allocate tasks to nodes. Experimental simulations were carried out to show the effectiveness of the approach. Obtained results were compared with a fog-oriented max–min algorithm and traditional max–min algorithm. The IoT-FCM moved users 38% closer to the fog node for fog-oriented max–min and 55% for the traditional max–min. While with respect to energy, IoT-FCM conserved an average of 150 KWh more energy versus the other algorithms. For the other part of the model, which is the terminal layer; IoT-FCM modified the LIBP protocol by adding multiple sinks. Performance evaluations were done using Cooja on Contiki and obtained results show that the modified LIBP with its use of multiple sink nodes, was more robust and tolerant to node failure and was also more energy conservatory. Of significant note in this work is that the two layers were simulated on different environments; therefore, the authors of this work, did not upload tasks/data directly from the terminal layer to the fog layer of the IoT-FCM model during testing. This could however be considered in future works.

## Figures and Tables

**Table 1:** Summary of related works.

The Works	Work Point	Deficiencies
Yang et al. [21]	They proposed a model that considers circuit, computation, offloading energy consumption to evaluate the overall energy efficiency (EE) in homogeneous fog networks.	The work only focused on the overall energy and did not consider the energy conversions across both the fog and terminal layers.
Pang et al. [22]	They proposed a latency-driven cooperative task computing in multi-user fog–radio access networks, which characterizes the trade off between communication and computing across multiple F-RAN (Fog radio access network) nodes.	They did take consider energy consumption problem in both fog layer and terminal layer.
Intharawijitr et al. [15]	In terms of the communication distance, they defined a mathematical model of a fog network and the important related parameters to clarify the computing delay and communication delay in fog architecture.	The work did not take into account the energy consumption of the whole model.
Ogawa et al. [23]	The authors presented a use case considering energy consumption measurements of RPL and CTP, and proposed metrics for several scenarios running both RPL and CTP.	The authors did not consider the routing protocol’s robustness and reliability.
Felici–Castell et al. [24]	The work focused on analysing different strategies to gather information from different topics. The trade-offs between the “always send” and “local buffer” methods are verified experimentally, which considering power consumption, lifetime, efficiency and reliability.	The reliability of the sink node(s) was not considered.
Machado et al. [25]	The authors proposed a routing protocol based on routing energy and link quality (REL). The end-to-end link quality estimation mechanism, residual energy and hop count are used to select routes to improve the reliability and energy efficiency of IoT applications. In addition, REL proposes an event-driven mechanism to provide load balancing and to avoid premature depletion of energy by nodes/networks.	Their work did not take into account the effect of different number of sink nodes.

**Table 2:** Performance configuration and computing power parameters (adapted from CloudSim).

Fog Nodes	Node1	Node2	Node3	Node4	Node5	Node6	Node7	Node8
Pes	2	4	2	4	2	2	2	2
Mips	550	300	650	350	750	800	850	900
Energy Cost	10	12	14	15	16	18	20	22
Coordinates	{10,10}	{10,40}	{10,70}	{40,10}	{70,10}	{70,40}	{70,70}	{60,80}

**Table 3:** Task coordinates.

Task No.	Coordinates	Task No.	Coordinates	Task No.	Coordinates	Task No.	Coordinates	Task No.	Coordinates
0	{93,31}	20	{36,75}	40	{73,06}	60	{53,49}	80	{61,21}
1	{32,96}	21	{44,23}	41	{77,45}	61	{52,12}	81	{62,96}
2	{14,11}	22	{31,23}	42	{77,89}	62	{54,11}	82	{64,11}
3	{52,21}	23	{35,23}	43	{72,34}	63	{12,63}	83	{62,48}
4	{50,21}	24	{36,21}	44	{70,21}	66	{10,21}	84	{63,90}
5	{43,90}	25	{33,90}	45	{73,90}	65	{53,93}	85	{67,53}
6	{10,61}	26	{31,21}	46	{77,62}	66	{58,34}	86	{84,70}
7	{96,59}	27	{36,59}	47	{76,59}	67	{50,61}	87	{64,10}
8	{39,83}	28	{49,83}	48	{76,78}	68	{51,24}	88	{63,46}
9	{71,34}	29	{34,51}	49	{11,34}	69	{42,83}	89	{12,37}
10	{23,31}	30	{43,31}	50	{83,31}	70	{43,51}	90	{13,14}
11	{22,96}	31	{32,96}	51	{92,96}	71	{44,67}	91	{39,57}
12	{24,11}	32	{14,11}	52	{96,75}	72	{44,11}	92	{17,11}
13	{23,83}	33	{59,39}	53	{92,21}	73	{49,87}	93	{52,31}
14	{20,21}	34	{54,52}	54	{95,24}	74	{44,59}	94	{50,61}
15	{23,90}	35	{43,90}	55	{93,90}	75	{53,12}	95	{44,23}
16	{28,95}	36	{10,61}	56	{90,61}	76	{40,61}	96	{13,71}
17	{26,59}	37	{96,59}	57	{45,32}	77	{49,56}	97	{95,69}
18	{66,66}	38	{57,74}	58	{99,83}	78	{49,83}	98	{32,53}
19	{28,45}	39	{75,23}	59	{68,21}	79	{41,34}	99	{63,31}

**Table 4:** Energy consumption.

	Energy Consumption of 5 Sink Nodes	Energy Consumption of 5 Sink Nodes	Energy Consumption of Three Sink Nodes	Energy Consumption of Two Sink Nodes	Energy Consumption of One Sink Node
Sink node 1:	4.55%	5.71%	3.18%	5.86%	6.60%
Sink node 2:	5.84%	3.86%	6.00%	4.27%	none
Sink node 3:	3.48%	3.40%	3.96%	none	none
Sink node 4:	3.35%	3.28%	none	none	none
Sink node 5:	3.93%	none	none	none	none
Average:	4.23%	3.92%	4.38%	5.07%	6.6%

**Table 5:** Fault-tolerance and robustness.

	5 Sink Nodes					4 Sink Nodes				3 Sink Nodes			2 Sink Nodes		1 Sink Nodes
Sink node number	1	2	3	4	5	1	2	3	4	1	2	3	1	2	1
Distance	2	3	0	2	2	2	1	3	2	1	3	0	2	2	3
Recovery time	15.18 s	none				18.76 s	none			27.32 s	none		34.81 s	none	infinity
Number of nodes	6	2	0	19	23	6	7	17	20	5	0	45	11	39	50

## References

[1] Bonomi F., Milito R., Natarajan P., Zhu J. (2014). Fog computing: A platform for internet of things and analytics. Big Data and Internet of Things: A Roadmap for Smart Environments.

[2] Calheiros R.N., Ranjan R., Beloglazov A., De Rose C.A., Buyya R. (2011). CloudSim: A toolkit for modeling and simulation of cloud computing environments and evaluation of resource provisioning algorithms. Softw. Pract. Exp..

[3] Chauhan S.S., Joshi R. A weighted mean time min-min max-min selective scheduling strategy for independent tasks on grid. Proceedings of the 2010 IEEE 2nd International Advance Computing Conference (IACC).

[4] Ngqakaza L., Bagula A. (2014). Least Path Interference Beaconing Protocol (LIBP): A Frugal Routing Protocol for The Internet-of-Things. IFIP Wired/Wireless Internet Communications WWIC.

[5] Bagula A., Djenouri D., Karbab E. Ubiquitous Sensor Network Management: The Least Beaconing Model. Proceedings of the 2013 IEEE 24th Annual International Symposium on Personal, Indoor, and Mobile Radio Communications (PIMRC).

[6] Bagula A., Djenouri D., Karbab E. (2013). On the Relevance of Using Interference and Service Differentiation Routing in the Internet-of-Things. Internet of Things, Smart Spaces, and Next Generation Networking.

[7] Bagula A., Mandava M., Bagula H. (2018). A Framework for Supporting Healthcare in Rural and Isolated Areas. J. Netw. Commun. Appl..

[8] Bagula A., Phillip A., Zodi G. (2016). Service-Aware Clustering: An Energy-Efficient Model for the Internet-of-Things. Sensors.

[9] Bagula A., Lubamba C., Mandava M., Bagula H., Zennaro M., Pietrosemoli E. Cloud Based Patient Prioritization as Service in Public Health Care. Proceedings of the ITU Kaleidoscope 2016.

[10] Mandava M., Lubamba C., Ismail A., Bagula A. Cyber-Healthcare for Public Healthcare in the Developing World. Proceedings of the 2016 IEEE Symposium on Computers and Communication (ISCC).

[11] Hong K., Lillethun D., Ramachandran U., Ottenwälder B., Koldehofe B. Mobile fog: A programming model for large-scale applications on the internet of things. Proceedings of the Second ACM SIGCOMM Workshop on Mobile Cloud Computing.

[12] Oueis J., Strinati E.C., Barbarossa S. The fog balancing: Load distribution for small cell cloud computing. Proceedings of the 2015 IEEE 81st Vehicular Technology Conference (VTC Spring).

[13] Yangui S., Ravindran P., Bibani O., Glitho R.H., Hadj-Alouane N.B., Morrow M.J., Polakos P.A. A platform as-a-service for hybrid cloud/fog environments. Proceedings of the 2016 IEEE International Symposium on Local and Metropolitan Area Networks (LANMAN).

[14] Abedin S.F., Alam M.G.R., Tran N.H., Hong C.S. A fog based system model for cooperative IoT node pairing using matching theory. Proceedings of the 2015 17th Asia-Pacific Network Operations and Management Symposium (APNOMS).

[15] Intharawijitr K., Iida K., Koga H. Analysis of fog model considering computing and communication latency in 5G cellular networks. Proceedings of the 2016 IEEE International Conference on Pervasive Computing and Communication Workshops (PerCom Workshops).

[16] Deng R., Lu R., Lai C., Luan T.H. Towards power consumption-delay tradeoff by workload allocation in cloud-fog computing. Proceedings of the 2015 IEEE International Conference on Communications (ICC).

[17] Sarkar S., Misra S. (2016). Theoretical modelling of fog computing: A green computing paradigm to support IoT applications. IET Netw..

[18] Ningning S., Chao G., Xingshuo A., Qiang Z. (2016). Fog computing dynamic load balancing mechanism based on graph repartitioning. China Commun..

[19] He X., Sun X., Von Laszewski G. (2003). QoS guided min-min heuristic for grid task scheduling. J. Comput. Sci. Technol..

[20] Gao Y., Rong H., Huang J.Z. (2005). Adaptive grid job scheduling with genetic algorithms. Futur. Gener. Comput. Syst..

[21] Yang Y., Wang K., Zhang G., Chen X., Luo X., Zhou M.T. (2018). MEETS: Maximal energy efficient task scheduling in homogeneous fog networks. IEEE Internet Things J..

[22] Pang A.C., Chung W.H., Chiu T.C., Zhang J. Latency-driven cooperative task computing in multi-user fog-radio access networks. Proceedings of the 2017 IEEE 37th International Conference on Distributed Computing Systems (ICDCS).

[23] Ogawa H.S., de Oliveira B.T., Rodrigues T.d.J., Albertini B., Margi C.B. Energy consumption and memory footprint evaluation of RPL and CTP in TinyOS. http://sbrt.org.br/sbrt2016/anais/ST02/1570270153.pdf.

[24] Felici-Castell S., Pérez-Solano J.J., Segura-Garcia J., García-Pineda M., Soriano-Asensi A. (2018). Experimental trade-offs between different strategies for multihop communications evaluated over real deployments of wireless sensor network for environmental monitoring. Int. J. Distrib. Sens. Netw..

[25] Machado K., Rosário D., Cerqueira E., Loureiro A., Neto A., de Souza J. (2013). A routing protocol based on energy and link quality for internet of things applications. Sensors.

[26] Davis L. (1991). Handbook of Genetic Algorithms.

[27] Liu J., Luo X.G., Zhang X.M., Zhang F., Li B.N. (2013). Job scheduling model for cloud computing based on multi-objective genetic algorithm. Int. J. Comput. Sci. Issues.

[28] Tawfeek M.A., El-Sisi A., Keshk A., Torkey F. An Ant Algorithm for cloud task scheduling. Proceedings of the International Workshop on Cloud Computing and Information Security (CCIS).

[29] Konak A., Coit D.W., Smith A.E. (2006). Multi-objective optimization using genetic algorithms: A tutorial. Reliab. Eng. Syst. Saf..

[30] Jang S.H., Kim T.Y., Kim J.K., Lee J.S. (2012). The study of genetic algorithm-based task scheduling for cloud computing. Int. J. Control Autom..

[31] Naha R.K., Garg S., Georgakopoulos D., Jayaraman P.P., Gao L., Xiang Y., Ranjan R. (2018). Fog Computing: Survey of trends, architectures, requirements, and research directions. IEEE Access.

[32] Dunkels A., Gronvall B., Voigt T. Contiki-a lightweight and flexible operating system for tiny networked sensors. Proceedings of the 29th Annual IEEE International Conference on Local Computer Networks.

[33] Sehgal A. (2013). Using the contiki cooja simulator. Comput. Sci. Jacobs Univ. Brem. Campus Ring.

